# 

**Published:** 1895-02

**Authors:** 


### CONTENTS.

ORIGINAL COMMUNICATIONS—
The History of Medicine and Surgery in Georgia .......... 705
Incontinence of Urine in Children, Treated with Atropia .......... 716
The Induction of Labor in Nephritis .......... 719
The Surgical Treatment of General Peritonitis Due to the Dissemination of Septic Products .......... 723
SOCIETY REPORTS—
Clinical Society of Maryland .......... 728
CORRESPONDENCE—
Diphtheria and Antitoxin .......... 731
Castration for Hypertrophy of the Prostate Gland .......... 733
EDITORIAL—
The Medical Boards .......... 737
Contract Service by Railway Surgeons .......... 738
One Way to Fame; or Dr. Morton and His "Letheon" .......... 741
SELECTIONS AND ABSTRACTS—
Eye, Ear, Nose, and Throat .......... 744
Practical Chemistry and Pharmacy .......... 747
Surgery .......... 751
MEDICAL ITEMS .......... 759
BOOK REVIEWS .......... 763
ADVERTISERS' INDEX TO ADVERTISEMENTS .......... xii
CLASSIFIED ADVERTISING INDEX .......... xxx
MEDICAL ITEMS—Continued .......... ix, x, x

